# Treatment recommendations to cancer patients in the context of FDA guidance for next generation sequencing

**DOI:** 10.1186/s12911-019-0743-x

**Published:** 2019-01-18

**Authors:** Grace K. Dy, Mary K. Nesline, Antonios Papanicolau-Sengos, Paul DePietro, Charles M. LeVea, Amy Early, Hongbin Chen, Anne Grand’Maison, Patrick Boland, Marc S. Ernstoff, Stephen Edge, Stacey Akers, Mateusz Opyrchal, Gurkamal Chatta, Kunle Odunsi, Sarabjot Pabla, Jeffrey M. Conroy, Sean T. Glenn, Hanchun T. DeFedericis, Blake Burgher, Jonathan Andreas, Vincent Giamo, Maochun Qin, Yirong Wang, Kazunori Kanehira, Felicia L. Lenzo, Peter Frederick, Shashikant Lele, Lorenzo Galluzzi, Boris Kuvshinoff, Carl Morrison

**Affiliations:** 1 Department of Medicine, Roswell Park Comprehensive Cancer Center, Buffalo, NY 14263 USA; 2 OmniSeq, Inc., Buffalo, NY 14203 USA; 3 Department of Pathology, Roswell Park Comprehensive Cancer Center, Buffalo, NY 14263 USA; 4 Center for Personalized Medicine, Roswell Park Comprehensive Cancer Center, Buffalo, NY 14263 USA; 5 Cancer Genetics and Genomics, Roswell Park Comprehensive Cancer Center, Buffalo, NY 14263 USA; 6 Division of Gynecologic Oncology, Roswell Park Comprehensive Cancer Center, Buffalo, NY 14263 USA; 7 Department of Radiation Oncology, Weill Cornell Medical College, New York, NY 10065 USA; 8 Sandra and Edward Meyer Cancer Center, New York, NY 10065 USA; 9 Université Paris Descartes/Paris V, Paris, France; 10 Department of Surgery, Roswell Park Comprehensive Cancer Center, Buffalo, NY 14263 USA

**Keywords:** Next generation sequencing, Comprehensive genomic profiling, FDA guidance, Physician treatment recommendations

## Abstract

**Background:**

Regulatory approval of next generation sequencing (NGS) by the FDA is advancing the use of genomic-based precision medicine for the therapeutic management of cancer as standard care. Recent FDA guidance for the classification of genomic variants based on clinical evidence to aid clinicians in understanding the actionability of identified variants provided by comprehensive NGS panels has also been set forth. In this retrospective analysis, we interpreted and applied the FDA variant classification guidance to comprehensive NGS testing performed for advanced cancer patients and assessed oncologist agreement with NGS test treatment recommendations.

**Methods:**

NGS comprehensive genomic profiling was performed in a CLIA certified lab (657 completed tests for 646 patients treated at Roswell Park Comprehensive Cancer Center) between June 2016 and June 2017. Physician treatment recommendations made within 120 days post-test were gathered from tested patients’ medical records and classified as targeted therapy, precision medicine clinical trial, immunotherapy, hormonal therapy, chemotherapy/radiation, surgery, transplant, or non-therapeutic (hospice, surveillance, or palliative care). Agreement between NGS test report targeted therapy recommendations based on the FDA variant classification and physician targeted therapy treatment recommendations were evaluated.

**Results:**

Excluding variants contraindicating targeted therapy (i.e., *KRAS* or *NRAS* mutations), at least one variant with FDA level 1 companion diagnostic supporting evidence as the most actionable was identified in 14% of tests, with physicians most frequently recommending targeted therapy (48%) for patients with these results. This stands in contrast to physicians recommending targeted therapy based on test results with FDA level 2 (practice guideline) or FDA level 3 (clinical trial or off label) evidence as the most actionable result (11 and 4%, respectively).

**Conclusions:**

We found an appropriate “dose-response” relationship between the strength of clinical evidence supporting biomarker-directed targeted therapy based on application of FDA guidance for NGS test variant classification, and subsequent treatment recommendations made by treating physicians. In view of recent changes at FDA, it is paramount to define regulatory grounds and medical policy coverage for NGS testing based on this guidance.

## Background

The Food and Drug Administration (FDA) has announced new mechanisms for regulatory approval of next generation sequencing (NGS) [1]. This includes a new pathway for approval of NGS tests for tumor profiling using the New York State Department of Health (NYSDOH) as a FDA third-party reviewer of in vitro diagnostics [2]. Central to this review is the fundamental tenet that NGS tests cover biomarkers with predictive value that change over time as clinical and scientific discoveries are made. As such, the FDA now recognizes three evidence levels that support the actionability and clinical utility of NGS tests [1]. Level 1 variants are defined by FDA as essential for the safe and effective use of a corresponding therapeutic. In seeking FDA approval, NGS tests may include level 1 evidence claims for a specific drug based on support for the analytical validity of the test for each specific biomarker and a clinical study that establishes either the link between the result of that test and patient outcomes or the clinical concordance to a previously approved companion diagnostic. Level 2 variants are defined by FDA as enabling physicians to use information about their patients in accordance with supporting clinical evidence, such as professional guidelines and/or peer-reviewed publications. Level 3 variants are defined by FDA as informational or used to direct patients toward clinical trials. Such claims are supported by analytical validation, principally through a representative approach when appropriate, and clinical or mechanistic rationale for inclusion in the panel, including peer-reviewed publications or in vitro preclinical models. While many groups have implemented other classification approaches for disease-biomarker-drug evidence associations, the FDA’s approach uniquely focuses on NGS and explicitly requires the analytical validity of the gene-variants tested.

Not unlike many other major cancer-focused medical academic centers [3–10], Roswell Park Comprehensive Cancer Center (Buffalo, NY) developed a precision oncology initiative that included NGS tests approved by the New York State Department of Health (NYSDOH). In 2016, an NGS pan-cancer comprehensive genomic profiling panel called OmniSeq Comprehensive® (OCP) (OmniSeq®, Buffalo, NY) was launched, based upon the Oncomine™ Comprehensive Assay by ThermoFisher Scientific (Carpinteria, CA). To evaluate the utility of the above described FDA variant classification guidance for oncologist treatment recommendations, we interpreted and applied the three-tier evidence schema to OCP test results from a large cohort of sequentially tested advanced cancer patients, and assessed agreement between NGS test targeted therapy recommendations and subsequent physician treatment recommendations for their patients.

## Methods

The OCP test uses tumor tissue to detect all classes of somatic genomic alterations in 144 cancer-associated genes. As seen in Table 1**,** the DNA-Seq component of the test detects somatic mutations (single nucleotide variants, insertions and deletions) and copy number variants in both oncogenes and tumor suppressor genes, while the RNA-Seq component performs rearrangement (fusion) analysis in oncogenes. DNA mutational analysis requires a minimum depth of 457 reads and uses a hot spot coverage strategy to detect gain-of-function mutations in oncogenes, while copy number analysis detects high level amplification. DNA mutational analysis also detects loss-of-function mutations in tumor suppressor genes using a complete coding sequence coverage strategy, while copy number analysis detects homozygous deletions. The RNA analysis detects fusions. The OCP test is approved for clinical use by New York State Clinical Laboratory Evaluation Program (NYS CLEP), which requires orthogonal confirmation by secondary technologies for somatic mutations. A proprietary bioinformatics pipeline filters single nucleotide polymorphisms and identifies reportable variants, including variants of unknown therapeutic significance (VUTS), based on pathogenicity using multiple public genomic content sources such as COSMIC, 1000 Genomes Project, dbSNP, SIFT, PolyPhen, and ClinVar.

**Table 1:** Gene-variants tested by OmniSeq Comprehensive®

Variant Type	Genes	Technology
Single Nucleotide Variants (SNVs), Insertions, Deletions, and Indels	Hotspot: ABL1, AKT1, ALK, AR, ARAF, BRAF, BTK, CBL, CDK4, CHEK2, CSF1R, CTNNB1, DDR2, DNMT3A, EGFR, ERBB2, ERBB3, ERBB4, ESR1, EZH2, FGFR1, FGFR2, FGFR3, FLT3, FOXL2, GATA2, GNA11, GNAQ, GNAS, HNF1A, HRAS, IDH1, IDH2, IFITM1, IFITM3, JAK1, JAK2, JAK3, KDR, KIT, KNSTRN, KRAS, MAGOH, MAP2K1, MAP2K2, MAPK1, MAX, MED12, MET, MLH1, MPL, MTOR, MYD88, NFE2L2, NPM1, NRAS, PAX5, PDGFRA, PIK3CA, PPP2R1A, PTPN11, RAC1, RAF1, RET, RHEB, RHOA, SF3B1, SMO, SPOP, SRC, STAT3, U2AF1, XPO1	DNA-Seq
	Full Coding: APC, ATM, BAP1, BRCA1, BRCA2, CDH1, CDKN2A, FBXW7, GATA3, MSH2, NF1, NF2, NOTCH1, PIK3R1, PTCH1, PTEN, RB1, SMAD4, SMARCB1, STK11, TET2, TP53, TSC1, TSC2, VHL, WT1	
Copy Number Gain	ACVRL1, AKT1, APEX1, AR, ATP11B, BCL2L1, BCL9, BIRC2, BIRC3, CCND1, CCNE1, CD274, CD44, CDK4, CDK6, CSNK2A1, DCUN1D1, EGFR, ERBB2, FGFR1, FGFR2, FGFR3, FGFR4, FLT3, GAS6, IGF1R	
Copy Number Loss	APC, ATM, BAP1, BRCA1, BRCA2, CDH1, CDKN2A, FBXW7, GATA3, MSH2, NF1, NF2, NOTCH1, PIK3R1, PTCH1, PTEN, RB1, SMAD4, SMARCB1, STK11, TET2, TP53, TSC1, TSC2, VHL, WT1	
Fusions	ABL1, AKT3, ALK, AXL, BRAF, EGFR, ERBB2, ERG, ETV1, ETV4, ETV5, FGFR1, FGFR2, FGFR3, MET, NTRK1, NTRK2, NTRK3, PDGFRA, PPARG, RAF1, RET, ROS1	RNA-Seq

Filtered, detected variants are submitted to a comprehensive knowledgebase of therapeutic associations, which determines whether or not there are therapeutic associations at the nucleotide, codon, exon, gene, or fusion level. A laboratory information engine with reporting rules specific to oncogenes and tumor suppressor genes to determine clinical significance in the final report. Variants in tumor suppressor genes must be pathogenic or deleterious by both SIFT and PolyPhen to be reported. While OCP does not sequence matching non-tumor tissue from tested patients, it is possible that germline mutations can be identified from tumor-only sequencing results without direct analysis of germline DNA. OCP reports detected mutations in genes prescribed by the American College of Medical Genetics and Genomics (ACMG) [11] as potentially hereditary, and directs physicians to further investigate by germline testing if clinically applicable. OCP test performance characteristics were analytically validated by OmniSeq Laboratories under the requirements of the Clinical Laboratory Improvement Amendments (CLIA) of 1988, and OmniSeq, Inc. is licensed by CLIA, College of American Pathologists (CAP), and the NYS CLEP to perform high-complexity molecular diagnostic testing. As such, OCP meets the analytical requirements put forth in the FDA variant classification guidelines for NGS testing. Additional details regarding OCP methodology, clinical validity, and performance characteristics can be found in the National Center for Biotechnology Information (NCBI) Genetic Testing Registry (https://www.ncbi.nlm.nih.gov/gtr/tests/552042/overview/).

Each gene-variant detected and reported by OCP tests previously performed between June 2017 to June 2017 (*n* = 657) was mapped to one or more levels of evidence based on interpretation of the FDA guidance for actionable variant classification, as follows: Level 1: variants listed on current FDA and/or European Medical Association (EMA) approved targeted therapy labels required for drug administration; Level 2: variants described in publicly available professional practice guidelines described as having evidence of response, resistance, or non-response to targeted therapeutics. Evidence sources to describe level 2 variants included professional practice guidelines established by the National Comprehensive Cancer Network (NCCN), and the European Society for Medical Oncology (ESMO). A complete list of level 1 and 2 therapeutic variant associations is shown in Table 2. Level 3: variants used as inclusion criteria or direct therapeutic targets of agents in active clinical development. Automated and manual review of investigational studies at https://clinicaltrials.gov was used for the identification of level 3 variants. As of this writing, there were 160 unique targeted agents, in use alone or in combination in over 350 recruiting precision medicine trials with OCP variants that act as direct therapeutic targets of the investigational agent, inclusion criteria, or both. Off-label use of variants listed on current FDA and/or EMA approved targeted therapy drug labels required for administration qualify were also defined as level 3 evidence since many targeted therapy trials are engaged in expanding existing indications to other tumor types.

**Table 2:** Level 1 and 2 evidence therapeutic variant associations tested by OmniSeq Comprehensive® (June 2017)

Level 1 (Companion Diagnostics)		
Genomic Variant(s)	Tumor Type	Therapy(ies)
ALK fusion	Lung	alectinib, brigatinib, ceritinib, crizotinib
BRAF V600E or BRAF V600K mutation	Melanoma	cobimetinib + vemurafenib; dabrafenib; dabrafenib + trametinib; trametinib; vemurafenib
BRAF V600E or BRAF V600K mutation	Lung	dabrafenib + trametinib
BRCA mutation	Ovarian	olaparib, rucaparib
EGFR exon 19 deletion	Lung	afatinib; bevacizumab + erlotinib; erlotinib; gefitinib; osimertinib
EGFR exon 20 insertion^a^	Lung	gefitinib
EGFR G719 mutation	Lung	afatinib; gefitinib
EGFR L858R mutation	Lung	afatinib; bevacizumab + erlotinib; erlotinib; gefitinib; osimertinib
EGFR L861Q mutation	Lung	afatinib; gefitinib
EGFR S768I mutation	Lung	afatinib; gefitinib
EGFR T790 M mutation^a^	Lung	gefitinib
EGFR T790 M mutation	Lung	osimertinib
ERBB2 amplification	Breast	ado-trastuzumab emtansine; lapatinib + aromatase inhibitor; lapatinib + chemo; lapatinib + trastuzumab; neratinib; pertuzumab + trastuzumab + chemo; trastuzumab; trastuzumab + aromatase inhibitor; trastuzumab + chemo
ERBB2 amplification	Esophageal	trastuzumab; trastuzumab + chemo
ERBB2 amplification	Gastric/GEJ	trastuzumab; trastuzumab + chemo
KRAS A146 or KRAS A59 mutation^a^	Colorectal	cetuximab; panitumumab
KRAS exon 2, 3 or 4 mutation^a^	Colorectal	cetuximab; cetuximab + chemo; panitumumab + chemo
KRAS G12, KRAS G13, KRAS K117, or KRAS Q61 mutation^a^	Colorectal	cetuximab; panitumumab
NRAS A146 or NRAS A59 mutation^a^	Colorectal	cetuximab; panitumumab
NRAS exon 2, 3 or 4 mutation^a^	Colorectal	cetuximab; cetuximab + chemo; panitumumab + chemo
NRAS G12, NRAS G13, NRAS K117, or NRAS Q61 mutation^a^	Colorectal	cetuximab; panitumumab
ROS1 fusion	Lung	crizotinib
Level 2 (Professional Practice Guidelines)		
Genomic Variant	Tumor Type	Therapy
ALK fusion	Sarcoma	ceritinib, crizotinib
ALK fusion^a^	Lung	EGFR tyrosine kinase inhibitor
BRAF mutation	Thyroid	vemurafenib
BRAF V600E mutation	Colorectal	cetuximab + vemurafenib + chemo; panitumumab + vemurafenib + chemo
BRAF V600E mutation	Lung	dabrafenib; vemurafenib
ERBB2 mutation	Lung	ado-trastuzumab emtansine
ERBB2 mutation	Head and Neck	trastuzumab
ERBB2 mutation	Breast	trastuzumab + chemo
KIT exon 9 mutation	Sarcoma	imatinib
KIT exon 11 mutation	Sarcoma	imatinib
KIT exon 11 or KIT exon 13 mutation	Melanoma	imatinib
KRAS mutation^a^	Lung	EGFR tyrosine kinase inhibitor (afatinib; erlotinib; erlotinib; gefitinib; osimertinib)
MET amplification or MET exon 14 skipping mutation	Lung	crizotinib
NF1 mutation^a^	Sarcoma	imatinib
PDGFRA D842V mutation^a^	Sarcoma	dasatinib; imatinib
RET fusion	Lung	cabozantinib; vandetanib
RET mutation	Thyroid	vandetanib
ROS1 fusion	Lung	alectinib; ceritinib
ROS1 fusion^a^	Lung	EGFR tyrosine kinase inhibitor (afatinib; erlotinib; erlotinib; gefitinib; osimertinib)

^a^These mutations confer resistance to the associated therapeutic (contraindication)

Age, gender, stage of disease, and tumor type were retrieved from test requisition data. Medical records were reviewed for patients with at least 60 days of post-test follow up time available in order to collect treatment status at the time of test order and physician treatment recommendations made for patients within 120 days after NGS results became available. Physicians’ post-test patient treatment recommendations were classified as targeted therapy, precision medicine clinical trial, immunotherapy, hormonal therapy, chemotherapy/radiation, surgery, transplant, or non-therapeutic (hospice, surveillance, or palliative care), and were compared to NGS report recommendations for agreement.

## Results

### Patients

Patients with 29 tumor types (Table 3) were tested, with breast cancer, colorectal carcinoma, lung carcinoma, melanoma, ovarian carcinoma, prostate cancer, and sarcoma being most common with each representing 5% or more of the total tests. Median age of all patients tested (64) and gender (55% males, 45% females) was within expectations for a cancer therapeutic management-focused test. Most patients had advanced (Stage III/IV) disease (540/657; 88%), but for a patient subset, stage was not reported when the NGS test was ordered (77/657; 12%). The majority of tests (76%; 497/657) were for patients currently undergoing treatment and/or had at least one prior treatment when the NGS test was ordered. Repeat testing was mostly restricted to lung cancer patients with resistance to prior targeted therapies.

**Table 3:** Tested patient characteristics (*n* = 646)

Age	
Median, yrs	64
Range, yrs	6–93
Gender, n (%)	
Female	359 (55%)
Male	298 (45%)
Stage, n (%)	
Stage I	9 (1.4%)
Stage II	11 (1.7%)
Stage III	54 (8%)
Stage IV	506 (77%)
Unknown	77 (12%)
Tumor Type, n (%)	
Bladder	4 (0.61%)
Brain	15 (2.3%)
Breast	34 (5%)
Cervical	4 (0.61%)
Colorectal	68 (10%)
Endocrine	3 (0.46%)
Endometrial	25 (3.8%)
Esophageal	13 (2%)
Eye	1 (0.2%)
Female Genital	1 (0.2%)
Gallbladder	3 (0.5%)
Head and Neck	4 (0.6%)
Kidney and Renal Pelvis	10 (1.5%)
Liver and Bile Duct	1 (0.15%)
Lung	205 (31%)
Melanoma	51 (8%)
Mesothelioma	4 (0.6%)
Neuroendocrine Tumors	14 (2.1%)
Non-Melanoma Skin	2 (0.3%)
Ovarian	44 (7%)
Pancreatic	9 (1.4%)
Prostate	34 (5%)
Sarcoma	87 (13%)
Small Intestine	3 (0.5%)
Stomach	3 (0.5%)
Testicular	1 (0.2%)
Thymic	3 (0.5%)
Thyroid	4 (0.6%)
Unknown Primary	7 (1%)
Prior systemic treatment	
Unknown	4 (0.6%)
0	156 (24%)
1+	497 (76%)

### NGS test results and physician treatment recommendations by FDA evidence level

A total of 2777 genomic alterations were identified in 657 tests with an average of 4.2 mutations per test. Frequently identified mutations were level 3 (1532; 55%), followed by VUTS (1045; 38%), level 2 (107; 4%), and level 1 (93; 3%).

#### Level 1 variants

A total of 92 variants with companion diagnostic level 1 supporting evidence as the most actionable result were detected in 14% (89/657) of tests as the most actionable evidence across 5 tumor types (Table 4). For the majority of these tests (94%; 84/89), a single genomic alteration was identified, with and no more than two level 1 variants reported in the remaining 5 tests. All tests with a level 1 variant had at least one other level 2 or level 3 alteration. The most frequent level 1 variants detected were identified in *KRAS* (colorectal cancer), *EGFR* (lung cancer), and *BRAF* (melanoma). Additional tumor types with at least one level 1 variant detected included breast and ovarian cancer.

**Table 4:** Frequency of detected variants by FDA level of supporting evidence (as of June 2017)

Tests with variants supported by level 1 evidence as the most actionable (*n* = 89)			
Gene	Tumor Type	Number of Detected Variants (*n* = 92)	Variants
KRAS	Colorectal	32	Codon 12, 13, 61, 146 single nucleotide variants
EGFR	Lung	17	L858R, T790 M, exon 19 deletions
BRAF	Melanoma	17	V600E, V600K
BRCA1	Ovarian	7	Multiple single nucleotide variants & indels*
BRAF	Lung	5	V600E
ALK	Lung	5	ALK (EML4) fusion
NRAS	Colorectal	3	Codon 12 & 61 single nucleotide variants
BRCA2	Ovarian	3	Multiple single nucleotide variants*
ERBB2	Breast	2	HER-2 amplification
ROS1	Lung	1	ROS1 (EZR) fusion
Tests with variants supported by level 2 evidence as the most actionable (*n* = 100)			
Gene	Tumor Type	Number of Detected Variants (*n* = 109)	Detected Mutations
KRAS	Lung	71	Codon 12, 13, 61, 146 single nucleotide variants & insertions
EGFR	Lung	9	Atypical exon 19 indels, exon 20 insertions, G719C, S768I, L861Q
MET	Lung	7	MET amplification and exon 14 skip
NF1	Sarcoma (GIST)	5	Multiple single nucleotide variants*
KIT	Melanoma	4	Multiple exon 11 single nucleotide variants
ERBB2	Lung	3	E365K, Y742_A745dup
KIT	Sarcoma (GIST)	2	Exon 9 and 11 single nucleotide variants
BRAF	Colorectal	2	V600E
RET	Thyroid	1	M918 T
RET	Lung	1	RET (KIF5B) fusion
KRAS	Colorectal	1	V14I
NRAS	Colorectal	1	Q61R
KIT	Sarcoma	1	V559D (exon 11)
ALK	Sarcoma	1	ALK (TPM3) fusion

**Fig. 1:**
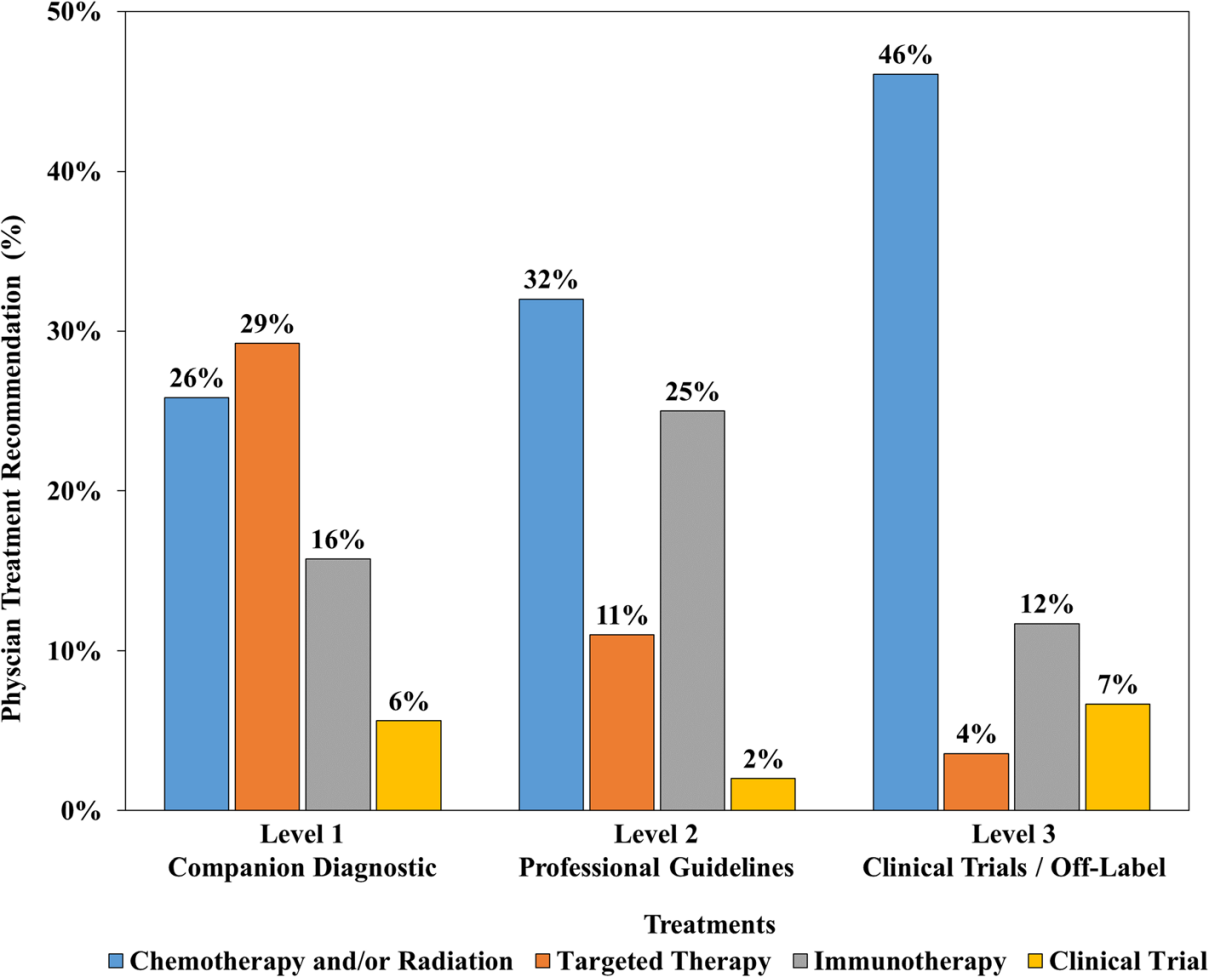
Physician treatment recommendations by the highest level of supporting clinical evidence to treat by targeted therapy for each test. Physician treatment recommendations for targeted therapies were most frequent for patient tests detecting variants supported by level 1 companion diagnostic evidence for targeted therapy (26/89; 29%), followed by tests with variants supported by level 2 practice guideline evidence as the most actionable result (11/100; 11%), and tests with variants supported by level 3 clinical trial/off-label evidence as the most actionable result (15/419; 4%). Recommendations for chemotherapy and/or radiation were more common for tests with variants supported by level 3 evidence (193/419; 46%) than for patient test results with level 2 (32/100; 32%) or level 1 (23/89; 26%) evidence. Recommendations for clinical trials were infrequent for test results across all 3 evidence levels for patients with companion diagnostic level 1 (5/89; 6%), level 2 (2/100; 2%), and level 3 (28/419; 7%) evidence. Recommendations for immunotherapy were relatively frequent for tests with targeted therapy level 1 (14/89; 16%), level 2 (25/100; 25%) or level 3 (49/419; 12%) supporting evidence

The most common physician treatment recommendation for tests with level 1 evidence variant results was targeted therapy for (26/89; 29%) of tests (48% excluding mutations with contraindications: *KRAS, NRAS*), followed by chemotherapy/radiation for 23%. The majority of these patients initiated chemotherapy/radiation prior to the test being performed with the presumed intention of planning for future treatment. Immunotherapy was a frequent recommendation (14/89; 16%), related to lack of *EGFR* or *ALK* alterations in NSCLC. Non-therapeutic recommendations (hospice or palliative care) were infrequent when variants with level 1 evidence were detected (2% of tests). Clinical trial recommendations were also uncommon (5/89; 6%) (Fig. 1).

#### Level 2 variants

A total of 109 variants with level 2 professional practice guideline supporting evidence for targeted therapy as the most actionable findings were detected in 100/657 (15%) of tests across five tumors types (colorectal carcinoma, lung cancer, melanoma, sarcoma, thyroid carcinoma). Similar to tests with a level 1 results, the majority of these tests (95/100; 97%) documented a single level 2 variant, and no more than two level 2 variants were reported for any given test. All tests with a level 2 variant also harbored at least one level 3 alteration. The most frequent level 2 variants were *KRAS* mutations in lung cancer. The second most frequent level 2 results encompassed atypical activating *EGFR* mutations in lung cancer patients, supporting use of EGFR inhibitors for certain alterations. The remaining level 2 variant results were distinct from level 1 results, and included a variety of alterations, all at a prevalence < 5% for that particular tumor type or a single case result (Table 4).

The most frequent physician treatment recommendation in the presence of level 2 evidence was chemotherapy/radiation (32/100; 32%), followed by immunotherapy for 25/100 tests (25%). Targeted therapy was a less frequent recommendation (11/98; 11%) with non-therapeutic recommendations being slightly more frequent (14/100; 14%). Clinical trial recommendations were also uncommon in this group (2/100; 2%) (Fig. 1).

#### Level 3 variants

There were 419 tests (65%) with level 3 genomic alterations as the most actionable variants. These results supported potential enrollment in either precision medicine clinical trials or use of off-label therapy, and were the most common result overall: mutations (1532/2777; 55%), tests (579/657; 88%), and all genes tested (67/144; 47%). Genes implicated by a level 3 mutation in at least 5% of all tests included *TP53*, *ATM*, *CDKN2A*, *BRCA2, PTEN*, *BRCA1*, *PIK3CA*, *TSC2*, *KDR*, *NF1, MET*, *RB1*, and *PTCH1* in descending order of prevalence (Table 5).

**Table 5:** Level 3 variant frequency by gene and number of tests

Gene	Number of Variants	Number of Tests	% of Tests
TP53	310	280	0.43
ATM	158	139	0.21
CDKN2A	96	91	0.14
BRCA2	101	90	0.14
PTEN	85	74	0.11
BRCA1	69	61	0.09
PIK3CA	63	56	0.09
TSC2	45	44	0.07
KDR	43	41	0.06
NF1	50	41	0.06
MET	37	37	0.06
RB1	37	37	0.06
PTCH1	37	34	0.05
STK11	30	28	0.04
FBXW7	25	25	0.04
NF2	24	24	0.04
NRAS	23	23	0.04
APC	23	22	0.03
TSC1	23	21	0.03
KRAS	19	18	0.03
IDH1	18	17	0.03
CDK4	18	17	0.03
CSF1R	15	14	0.02
EGFR	14	12	0.02
BRAF	13	11	0.02
ERBB2	15	11	0.02
FGFR3	12	10	0.02
FGFR1	10	9	0.01
AKT1	8	8	0.01
SMO	9	8	0.01
MSH2	9	6	0.01
CCND1	6	6	0.01
KIT	7	6	0.01
PIK3R1	6	6	0.01
RET	6	6	0.01
SMARCB1	5	4	0.01
CHEK2	4	4	0.01
CCNE1	4	4	0.01
MYC	4	4	0.01
BAP1	4	4	0.01
ERBB4	3	3	<.01
DDR2	3	3	<.01
FLT3	3	3	<.01
MAP2K1	3	3	<.01
ALK	3	3	<.01
ESR1	2	2	<.01
GNA11	2	2	<.01
ERBB3	2	2	<.01
HRAS	2	2	<.01
ABL1	2	2	<.01
JAK2	2	2	<.01
MTOR	2	2	<.01
MYCL	2	2	<.01
NFE2L2	2	2	<.01
NTRK1	2	2	<.01
MAPK1	2	2	<.01
AR	1	1	<.01
CDK6	1	1	<.01
FGFR2	1	1	<.01
JAK1	1	1	<.01
WT1	1	1	<.01
MDM2	1	1	<.01
NTRK3	1	1	<.01
RAF1	1	1	<.01
SRC	1	1	<.01
IDH2	1	1	<.01

For the 419 tests for which level 3 variants were identified as the most actionable, the rate of clinical trial treatment recommendations by physicians was 7% (28/419). Commonly recommended in this group was chemotherapy/radiation (193/419; 46%), followed by immunotherapy (49/419; 11%). Targeted therapy recommendations were uncommon (15/419; 4%). Non-therapeutic options were recommended to 10% (41/419) of the cases belonging to this group, and not remarkably different from patients with a level 2 result (14%) (Fig. 1).

Overall, a total of 111 mutations with off-label indications were identified in 90 tests with no level 1 or 2 evidence. The majority of these off-label indications were *BRCA1* or *BRCA2* mutations (90/111; 81%), which were documented across 20 different tumor types. Less frequent mutations linked to off-label indications included activating single nucleotide variants or indels (insertions/deletions) in *ERBB2* (HER-2), *KIT*, *BRAF*, and *RET*, as well as *MET* copy number gain or exon 14 skip. Off-label targeted therapy recommendations were infrequent (11/111; 10%).

#### VUTS

For a subset of tests (49/657; 7%), no variants were classified as having clinical significance based on FDA variant classification guidance. It is worthy to note that this group had the highest recommendation rates for chemotherapy/radiation (26/49; 53%), as well as and non-therapeutic options (8/49; 16%) compared to tests with variants supported by level 1, 2 or 3 evidence. Targeted therapy was never recommended in this group.

### Tumor type-specific results

Of 29 tumor types tested, 24 did not harbor level 1 or 2 variants. These included mesotheliomas, sarcomas, bladder, brain, cervical, endocrine, endometrial**,** gastroesophageal, eye, genital, gallbladder, head and neck, kidney, renal pelvis, liver, bile duct, neuroendocrine, non-melanoma skin, pancreatic, prostate, small bowel, stomach, testicular, thymic, thyroid, and unknown primary cancers. Most of these tumor types also did not have biomarker-directed indications approved for targeted therapy, either on label or in professional practice guidelines. None of the 34 prostate cancers tested expressed a level 1 or 2 alteration. Of the 24 tumor types with no actionable level 1 or 2 variants, only 2 (gastroesophageal carcinomas and gastrointestinal stromal tumors) harbored mutations that met the list of genomic variants identified as therapeutic association with a high level of evidence (Table 4).

The remaining 5 tumor types with at least one level 1 or 2 variant included melanoma, breast, colorectal, lung, and ovarian cancers accounted for 61% (402/657) of the total tests performed, and do have biomarker-directed indications for targeted therapy, accurately reflecting the prevalence of these tumors and their current position in the precision medicine hierarchy per oncologist test usage.

## Discussion

This study demonstrates the application of FDA-guidance for NGS variant classification in oncology therapeutic management, and compares the test results to treatment recommendations the way healthcare providers typically do (i.e., retrospectively) to obtain reimbursements. In particular, this study provides evidence from a current clinical practice where physicians formulate treatment recommendations based on a cancer-focused comprehensive NGS assay in the context of new guidelines from FDA. One conclusion from this study is that physicians do not recommend (or at least do not document recommending) clinical trials as often as they are indicated by NGS testing. We recognize that capturing treating physicians clinical trial recommendations solely by medical record review may generate an underestimation of the actual physician intent (i.e. patient ineligibility based on pre-screening evaluation) or patient-physician interaction in which the highest level of evidence is associated with an investigational study (level 3 evidence). In the group of tests with no on-label indications for targeted therapy, chemotherapy/radiation were the most frequent recommendations, suggesting that traditional approaches to cancer are still highly regarded by both oncologists and patients, or at least are still regarded as the most viable option given all the complex factors that contribute to decision making. This study also suggests that physicians are using NGS results appropriately to recommend targeted therapy. Targeted therapy was recommended for 48% of tests with level 1 evidence, 11% of tests with level 2 evidence, 3.6% of tests with level 3 evidence, and never in the presence of VUTS. This may have been positively influenced by the fact that testing and recommendations were performed in a NCI-designated comprehensive cancer center with access to a molecular tumor board, which can aid in treatment decision recommendations.

The majority of tumor types tested in this study have no level 1 or 2 therapeutic drug associations, implying that maximal benefit could originate from either a clinical trial or an off-label recommendation, both of which were infrequently recommended by treating physician, at least during the data collection period in this study. There is doubt that following such patients in a registry will provide any benefit when physicians do not strongly support enrollment in clinical trials but generally recommend chemotherapy/radiation. One factor that may offset such a perceived lack of benefit from NGS testing for targeted therapy is immunotherapy. Immunotherapy was recommended by physicians in 16% of tests with level 1 evidence, 25% of tests with level 2 evidence, 12% of tests with level 3 evidence, and 6% of tests documenting VUTS. This suggests that physicians are often using NGS results to exclude targeted therapy prior to recommending immunotherapy.

Many centers in the US are implementing clinical oncology pathways and collecting evidence as we did, which may be more accurate and representative than the current approach of multiple laboratories. Such pathways should aim at aligning care to national guidelines or, when appropriate, define the rationale for deviation from guidelines [12]. Another key objective for these pathways should be to support clinical trials, and to identify the barriers to enrollment. Some pathway programs require review of relevant clinical trials as the first treatment choice before allowing selection of “standard” therapies [13]. Pathway systems also provide a registry that identifies patients starting a new cancer treatment, what that treatment is, if the patient accepted to join a clinical trial, and how treatment compares to national guidelines. Pathway systems may provide the basis for proper identification of patients eligible for NGS and related clinical trials. Databases generated in the context of these pathways may also provide a robust foundation for the required National Institute of Health Genetic Testing Registry (NIH GTR) as per the new FDA guidelines for NGS.

There are multiple limitations to the present study, many of which related to the fact that it was retrospective and involved no direct interaction with physicians other than test ordering, test reporting, or molecular tumor board review. Recommendations were captured from clinical documentation which may be incomplete (e.g. not specifically documenting that a patient with is not eligible for clinical trial due to exclusion criteria) and may not record past discussions with ordering physicians. This may partially explain the low documented rate of recommendations to clinical trials, which may not fully represent the actual clinical practice. Eligibility for a clinical trial often includes multiple factors beyond molecular parameters, such as number and types of prior therapies, comorbidities, etc. Physician recommendations were captured within 120 days post-NGS test for patients that had at least 60 days of follow up time. Several clinical trials, such as the NCI-MATCH, require that patients have explored standard therapeutic options first and recommendation for a clinical trial may occur much later in a patient’s treatment course, often after multiple episodes of care.

## Conclusions

NGS is a well-established technology, yet it lacks standardized regulatory approval. The majority of tumors do not have on-label, variant-directed targeted therapy indications, however, our data show that physicians generally understand NGS results and apply them appropriately, and that the FDA has provided valid guidance a pathway for medical policy coverage for at least a subset of tumor types. As precision medicine advances toward regulatory approval in the standard of care setting, coverage by both the Centers for Medicaid and Medicare (CMS) and other payers will require a consistent reproducible method of presenting evidence.

## Abbreviations

ACMG: American College of Medical Genetics and Genomics
CAP: College of American Pathologists
CLIA: Clinical Laboratory Improvement Amendments
EMA: European Medical Association
ESMO: European Society for Medical Oncology
FDA: Food and Drug Administration
GTR: Genetic Testing Registry
NCBI: National Center for Biotechnology Information
NCCN: National Comprehensive Cancer Network
NCI-MATCH: National Cancer Institute’s Molecular Analysis for Therapy Choice.
NGS: Next generation sequencing
NIH: National Institute of Health
NYS CLEP: New York State Clinical Laboratory Evaluation Program
NYSDOH: New York State Department of Health
OCP: OmniSeq Comprehensive®
RPCCC: Roswell Park Comprehensive Cancer Center
